# Epidemiology of human parechovirus, Aichi virus and salivirus in fecal samples from hospitalized children with gastroenteritis in Hong Kong

**DOI:** 10.1186/1743-422X-11-182

**Published:** 2014-10-18

**Authors:** Cyril C Y Yip, Kin-Land Lo, Tak-Lun Que, Rodney A Lee, Kwok-Hung Chan, Kwok-Yung Yuen, Patrick C Y Woo, Susanna K P Lau

**Affiliations:** Department of Microbiology, The University of Hong Kong, Hong Kong, Hong Kong; State Key Laboratory of Emerging Infectious Diseases, The University of Hong Kong, University Pathology Building, Queen Mary Hospital, Hong Kong, Hong Kong; Research Centre of Infection and Immunology, The University of Hong Kong, Hong Kong, Hong Kong; Carol Yu Centre for Infection, The University of Hong Kong, Hong Kong, Hong Kong; Department of Pathology, Tuen Mun Hospital, Hong Kong, Hong Kong; Department of Pathology, Pamela Youde Nethersole Eastern Hospital, Hong Kong, Hong Kong

**Keywords:** Human parechovirus, Aichi virus, Salivirus, Gastroenteritis, Fecal, children, Hong Kong

## Abstract

**Background:**

Emerging human picornaviruses, including human parechovirus (HPeV), Aichi virus (AiV) and salivirus (SalV) were found to be associated with gastroenteritis, but their roles in enteric infections are not fully understood. In addition, no report on the circulation of these viruses in Hong Kong is available. The objective of this study was to investigate the prevalence and genetic diversity of HPeV, AiV and SalV in fecal samples from hospitalized children with gastroenteritis in Hong Kong.

**Methods:**

Fecal samples from hospitalized children with gastroenteritis were subject to detection of HPeV, AiV and SalV by RT-PCR using consensus primers targeted to their 5′UTRs. Positive samples were subject to capsid and/or 3CD region analysis for genotype determination. The epidemiology of HPeV, AiV and SalV infections was analyzed.

**Results:**

Among 1,708 fecal samples subjected to RT-PCR using primers targeted to 5′UTR of HPeV, AiV and SalV, viruses were detected in 55 samples, with 50 positive for HPeV only, 3 positive for AiV only, 1 positive for both HPeV and AiV, and 1 positive for both HPeV and SalV. Phylogenetic analysis of the partial VP1 gene of the 33 HPeV strains revealed the presence of genotypes of HPeV- 1, 3, 4, 5, 7, 10, among which HPeV-1 was the predominant genotype circulating in our population. The peak activity of HPeV infection was in fall. Of the 3 children with AiV infection, the 3 AiV strains were found to belong to genotype A based on the phylogenetic analysis of their partial VP1 and 3CD regions. The genotype of a SalV strain detected in this study could not be determined. Co-detection of different pathogens was observed in 24 samples (43.6%) of 55 fecal samples positive for HPeV, AiV and SalV.

**Conclusions:**

HPeV, AiV and SalV were detected in fecal samples of hospitalized children with gastroenteritis in Hong Kong, with the former having the highest prevalence. HPeV-1 was the predominant genotype among HPeVs, while genotype A was the predominant genotype among AiVs in this study.

## Background

Picornaviruses are single-stranded positive-sense RNA viruses that can cause a variety of diseases in humans and animals. Based on genotypic and serological characterization, picornaviruses are divided into 26 genera, including *Aphthovirus*, *Aquamavirus*, *Avihepatovirus*, *Avisivirus, Cardiovirus*, *Cosavirus*, *Dicipivirus*, *Enterovirus*, *Erbovirus*, *Gallivirus*, *Hepatovirus*, *Hunnivirus*, *Kobuvirus*, *Megrivirus*, *Mischivirus, Mosavirus, Oscivirus*, *Parechovirus*, *Pasivirus, Passerivirus, Rosavirus, Salivirus*, *Sapelovirus*, *Senecavirus*, *Teschovirus* and *Tremovirus* (http://www.picornaviridae.com/). In the past few years, there has been a dramatic increase in the number of novel picornaviruses identified and genome sequenced [1–12]. Picornaviruses are also well known for their ability to undergo mutations and recombination, which may lead to the emergence of novel genotypes associated with increase virulence [13–16].

Diarrhea is one of the leading causes of death in the world [17], especially for children below 5 years of age. Since around 40% of the cases remain undiagnosed [18], research has been conducted to identify unrecognized causative agents. Recent advanced molecular techniques have allowed the discovery of novel viruses including picornaviruses from patients with gastroenteritis [2, 18–22]. Human parechoviruses (HPeVs) have been classified into 16 types (http://www.picornaviridae.com/parechovirus/hpev/hpev.htm), in which types 1, 3–6, 8, 10 and 11 were found to be associated with gastroenteritis [23–28]. HPeVs have been reported in fecal samples from patients with gastroenteritis in various parts of the world, suggesting that the viruses are circulating worldwide [23–28]. Another picornavirus, Aichi virus (AiV), which belongs to the genus *Kobuvirus*, was first isolated from a patient with oyster-associated gastroenteritis by BS-C-1 cell culture in Japan in 1989 [29] and its complete genome sequence was determined in 1998 [21]. Epidemiological studies on AiV have demonstrated a global distribution of this virus [30–37]. Based on phylogenetic analysis of the sequences between the C-terminus of 3C and the N-terminus of 3D, AiV isolates were divided into 3 genotypes: A, B and a newly proposed genotype C [30, 33, 36, 38]. Most recently, salivirus (SalV) or klassevirus, which belongs to the genus *Salivirus*, was identified in pediatric stool samples from patients with diarrhea [2, 39, 40]. Based on the results from genomic characterization, SalV was most closely related to, but distinct from other members of the genus *Kobuvirus*. To date, only few studies reported the molecular epidemiology of SalV infection [40–42].

Although previous findings revealed the existence of HPeV, AiV and SalV, the roles of these viruses in enteric infections are not fully understood. In addition, no report on the circulation of these viruses in Hong Kong is available. Therefore, a molecular epidemiological study was conducted to investigate the prevalence and genetic diversity of these viruses in the fecal samples from pediatric patients with gastroenteritis in Hong Kong by reverse transcription-polymerase chain reaction (RT-PCR) using consensus primers targeted to their 5′ untranslated regions (5′UTRs). Phylogenetic analysis of other gene regions (capsid or 3CD region) was performed to determine the genotype of the HPeV, AiV and SalV.

## Results

### Detection of HPeV, AiV and SalV in fecal samples from pediatric patients with acute gastroenteritis

**Figure 1:**
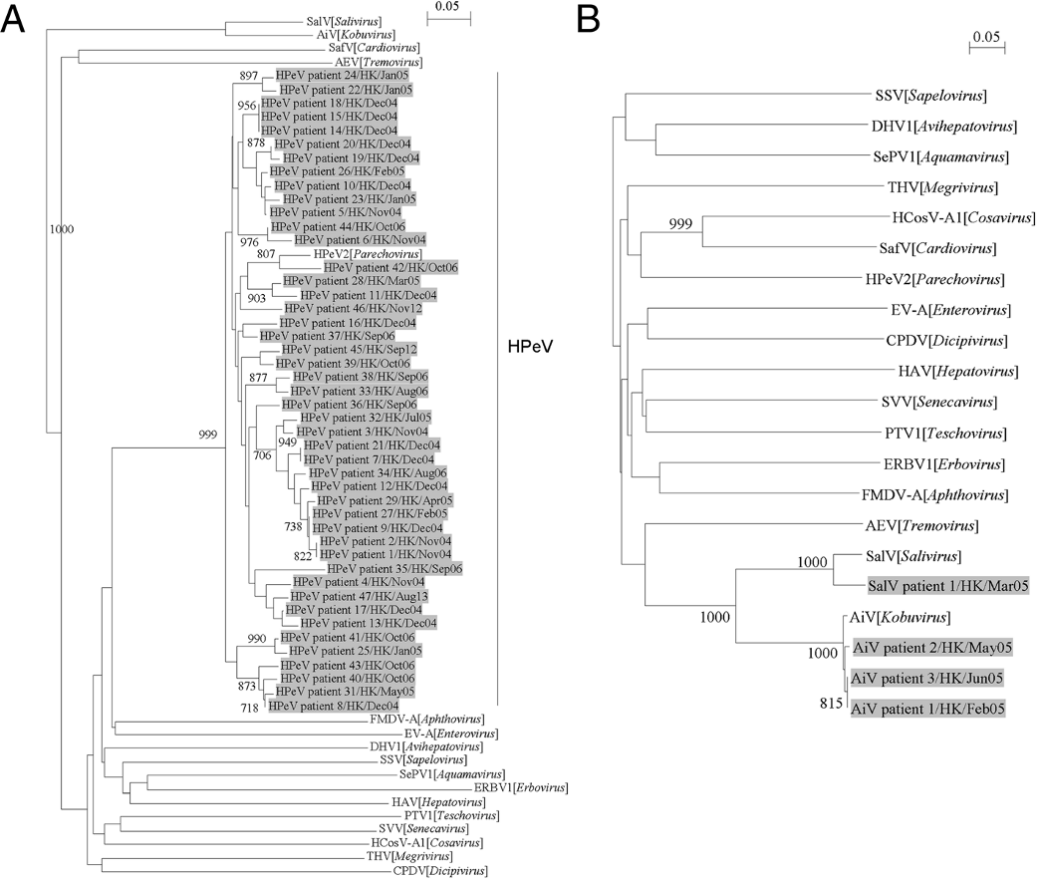
**Phylogenetic analysis of partial 5′UTR sequences of (A) HPeV strains from 47 patients, (B) 3 AiV and 1 SalV strains from 4 patients.** The trees were inferred from 5′UTR data by the neighbor-joining method, with bootstrap values calculated from 1000 trees. **(A)** 145 and **(B)** 188 nucleotide positions in each 5′UTR were included in the analysis. The scale bar indicates the estimated number of substitutions of 20 nucleotides as indicated. The virus strains detected in this study are highlighted in grey. Virus abbreviations (GenBank accession numbers shown in parentheses): AEV, avian encephalomyelitis virus (NC_003990); AiV, Aichi virus (NC_001918); CPDV, canine picodicistrovirus (JN_819202); DHV1, duck hepatitis virus 1 (NC_008250); ERBV1, equine rhinitis B virus 1 (NC_003983); FMDV-A, foot-and-mouth disease virus-A (JQ_082983); HAV, hepatovirus (NC_001489); HCosV-A1, human cosavirus A1 (FJ_4389020); EV-A, human enterovirus A (AY_697458); HPeV2, human parechovirus type-2 (AJ_005695); PTV1, porcine teschovirus 1 (NC_003985); SafV, saffold virus (EF_165067); SalV, salivirus (GQ_179640); SePV1, seal picornavirus 1 (NC_009891); SSV, simian sapelovirus (AY_064708); SVV, Seneca Valley virus (NC_011349); THV, turkey hepatitis virus (HQ_189775).

One thousand four hundred and forty fecal samples (retrospective study period) and 268 fecal samples (prospective study period) from hospitalized children with gastroenteritis were screened for the presence of HPeV, AiV and SalV by RT-PCR using primers targeted to their corresponding 5′UTR. RT-PCR for HPeV, AiV and SalV was positive in 55 samples from 49 patients, among which 52 (3.4% for retrospective, 1.1% for prospective) from 47 patients contained HPeV, 4 (0.28% for retrospective) from 3 patients contained AiV (1 co-detected in a sample with HPeV strain patient 30/HK/May05) and 1 (0.07% for retrospective) contained SalV (co-detected in a sample with HPeV strain patient 28/HK/Mar05) by sequencing and phylogenetic analysis (Figure 1). Among the 6 patients with multiple detections, the sequences of the picornavirus strains persistently shed from the same patient were identical.

**Figure 2:**
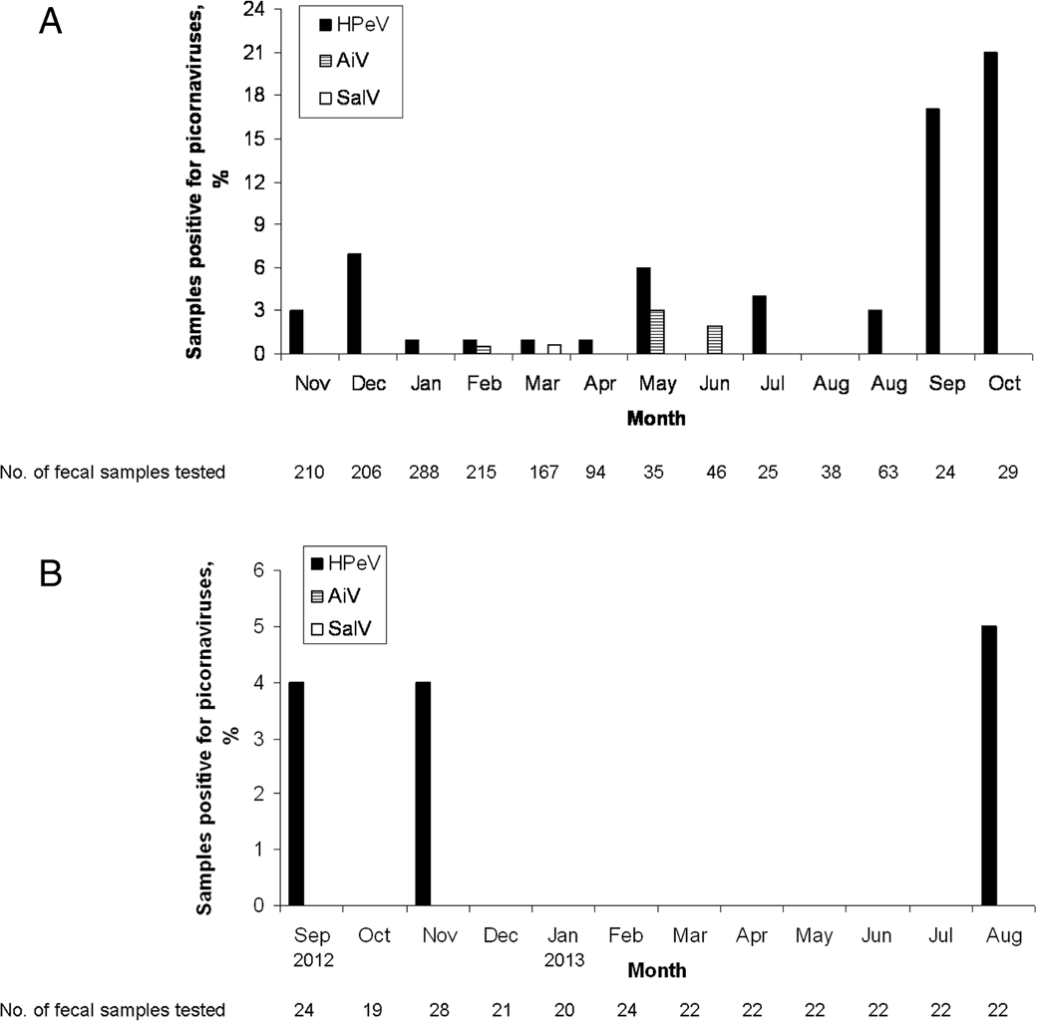
**Seasonality distribution of HPeV, AiV and SalV infections in (A) November 2004 to August 2005 and August 2006 to October 2006 and (B) September 2012 to August 2013.**

The characteristics of the 49 patients with HPeV, AiV and SalV detected in fecal samples were summarized in Table 1. The median age of the 47 patients with HPeV was 17 months (range, 2 months - 8 years). Thirty-three were male and 14 were female. HPeV was detected nearly throughout the year in the retrospective period (November 2004 to August 2005, and August to October 2006), with the highest detection frequency in fall (Figure 2), while 3 cases of HPeV infection were identified in September and November 2012, and in August 2013. HPeV was repeatedly detected in separate fecal samples from 5 patients (patient 3, 19, 22, 23, and 33). Diarrheal pathogens were frequently found in the HPeV-positive fecal samples, with rotavirus identified in 8, norovirus in 1, human bocavirus (HBoV) in 2, AiV in 1, SalV in 1, *Salmonella enterica* serotypes in 5, *Campylobacter jejuni* in 1, *Staphylococcus aureus* in 7, enteropathogenic *Escherichia coli* in 1, and *Aeromonas* species in 2.

**Table 1:** **Clinical characteristics and demographic data of the 49 patients with HPeV, AiV and SalV detected in fecal samples**

Patient	Month of detection	Sex	Age	Virus identified in this study	Other pathogens detected from fecal samples	Multiple detections (days apart)
1	Nov 2004	M	3	HPeV	None	ND
2	Nov 2004	M	4 m	HPeV-1	None	ND
3	Nov 2004	F	10 m	HPeV-1	None	Yes (0)
4	Nov 2004	M	2	HPeV-1	Rotavirus	ND
5	Nov 2004	M	2 m	HPeV-1	HBoV	ND
6	Nov 2004	M	6 m	HPeV-1	*S. aureus*	ND
7	Dec 2004	F	1	HPeV	None	ND
8	Dec 2004	M	7 m	HPeV-3	*S. aureus*, HBoV	ND
9	Dec 2004	F	5 m	HPeV-1	*S. aureus*, rotavirus	ND
10	Dec 2004	M	2	HPeV-1	Rotavirus	ND
11	Dec 2004	M	6 m	HPeV	None	ND
12	Dec 2004	F	10 m	HPeV	Rotavirus	ND
13	Dec 2004	M	2	HPeV-1	None	ND
14	Dec 2004	F	1	HPeV-1	None	ND
15	Dec 2004	M	1	HPeV	None	ND
16	Dec 2004	F	6 m	HPeV	*S. aureus*	ND
17	Dec 2004	M	5 m	HPeV-1	None	ND
18	Dec 2004	F	3	HPeV-1	Rotavirus	ND
19	Dec 2004	M	1	HPeV-1	None	Yes (15)
20	Dec 2004	M	8 m	HPeV-1	None	ND
21	Dec 2004	M	6 m	HPeV	*S. aureus*	ND
22	Jan 2005	F	1	HPeV-7	None	Yes (0)
23	Jan 2005	F	5 m	HPeV	None	Yes (26)
24	Jan 2005	M	5 m	HPeV-7	Rotavirus	ND
25	Jan 2005	M	9 m	HPeV-1	None	ND
26	Feb 2005	M	6 m	HPeV-1	None	ND
27	Feb 2005	M	5 m	HPeV-1	None	ND
28	Mar 2005	F	8	HPeV-4, SalV	None	ND
29	Apr 2005	F	1	HPeV-1	*Salmonella* group B, *Campylobacter jejuni*	ND
30	May 2005	M	2	HPeV-4, AiV	*Salmonella* group B	ND
31	May 2005	M	9 m	HPeV-3	*Aeromonas sobria*	ND
32	Jul 2005	M	1	HPeV-5	Enteropathogenic *E. coli*	ND
33	Aug 2006	F	7 m	HPeV-4	None	Yes (5)
34	Aug 2006	M	10 m	HPeV-1	*S. aureus*	ND
35	Sep 2006	M	3	HPeV-1	None	ND
36	Sep 2006	M	8 m	HPeV-5	*Salmonella* group D	ND
37	Sep 2006	F	1	HPeV-1	*Salmonella* group C	ND
38	Sep 2006	M	3	HPeV	None	ND
39	Oct 2006	M	1	HPeV-10	Rotavirus	ND
40	Oct 2006	M	1	HPeV	*S. aureus*, *Salmonella* group B	ND
41	Oct 2006	F	5 m	HPeV-1	None	ND
42	Oct 2006	M	3	HPeV	None	ND
43	Oct 2006	M	6 m	HPeV	None	ND
44	Oct 2006	M	2 m	HPeV-1	Rotavirus	ND
45	Sep 2012	M	7	HPeV-1	*Aeromonas veronii biovar sobria*	ND
46	Nov 2012	M	9 m	HPeV	Norovirus	ND
47	Aug 2013	M	7	HPeV	None	ND
48	Feb 2005	F	7	AiV	None	Yes (0)
49	Jun 2005	F	8 m	AiV	None	ND

HBoV, human bocavirus; *E. coli*, *Escherichia coli*; *S. aureus*, *Staphylococcus aureus*; ND, not done.

The median age of the 3 patients with AiV was 2 years (age range being 8 months - 7 years; M:F =1:2). The 4 AiV positive samples were detected in late winter and early summer in 2005 (Table 1). AiV was detected in a 7-year-old girl (patient 48) in 2 separate fecal samples collected on the same day. Co-detection of HPeV was found in an AiV-positive sample.

For SalV, the virus was detected in the fecal sample from an 8-year-old girl (patient 28) in March 2005. This fecal sample was co-detected with HPeV. No AiV and SalV were detected in fecal samples collected during September 2012 - August 2013.

### Genotyping of HPeV, AiV and SalV strains

**Figure 3:**
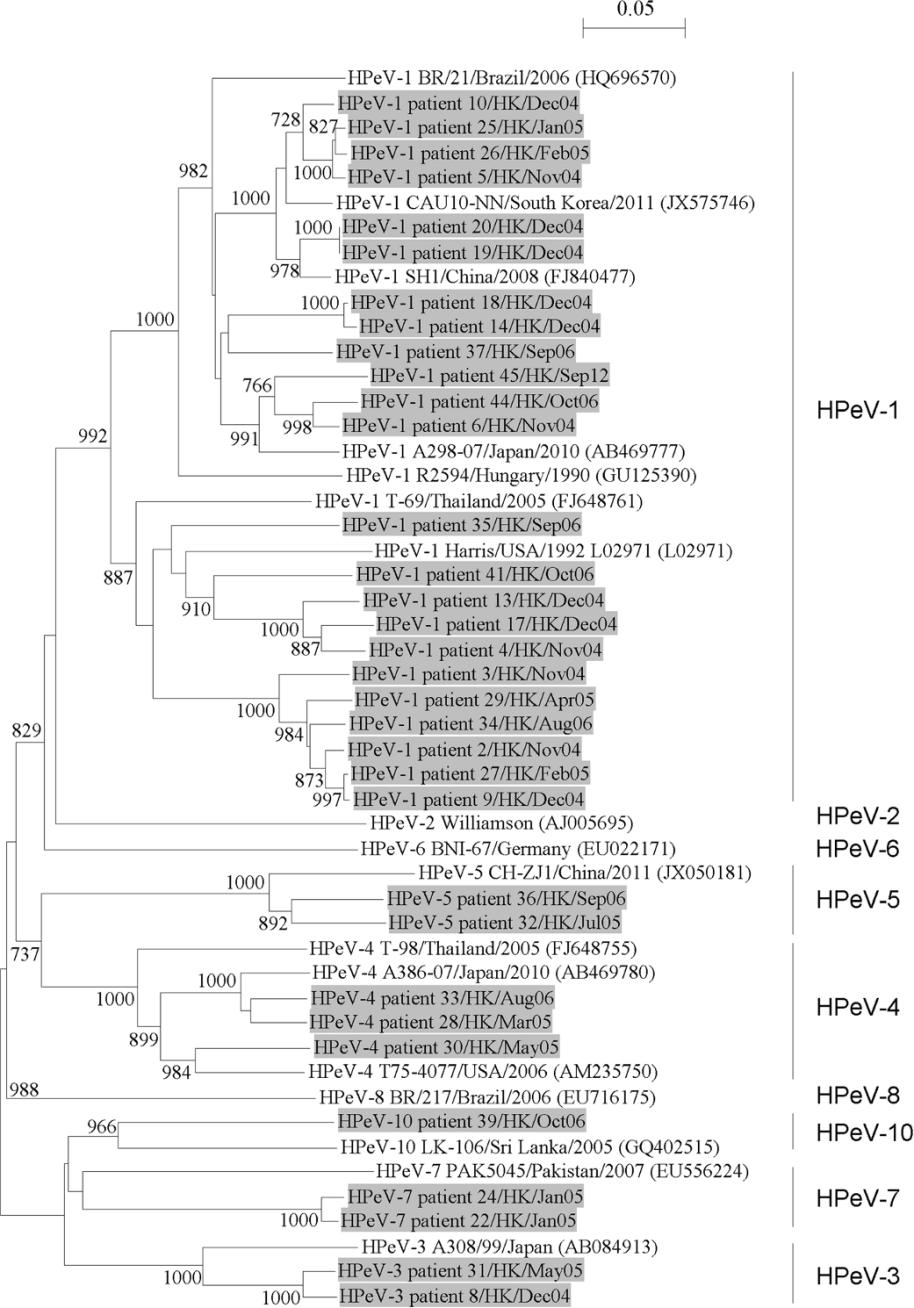
**Phylogenetic analysis of the partial VP1 capsid gene of the 33 HPeV strains.** The trees were constructed by the neighbor-joining method, with bootstrap values calculated from 1000 trees. Sequence for 628 nucleotide positions in VP1 gene was included in the analysis. The scale bar indicates the estimated number of substitutions of 20 nucleotides as indicated. The HPeV strains detected in the present study are highlighted in grey.

To determine the genotype of the HPeV, AiV and SalV strains detected in this study, amplification and sequencing of their partial VP1 capsid gene and/or 3CD region were performed. For HPeV, partial VP1 gene of 33 HPeV strains was successfully amplified and sequenced. A phylogenetic tree using nucleotide sequences of the partial VP1 gene of HPeV strains detected in Hong Kong and other HPeV strains with VP1 gene sequences available in GenBank was constructed (Figure 3). The 33 HPeV strains detected in the present study consisted of 6 types, including type 1 (23 strains), type 3 (2 strains), type 4 (3 strains), type 5 (2 strains), type 7 (2 strains) and type 10 (1 strain), indicating that HPeV-1 was the predominant genotype circulating in our population. Among the 5 HPeV-positive patients with multiple detections by RT-PCR targeted to 5′UTR, the partial VP1 gene of the strains shed from 2 of these patients could be amplified and the partial VP1 sequences of the HPeV strains from the same patient were found to be identical.

**Figure 4:**
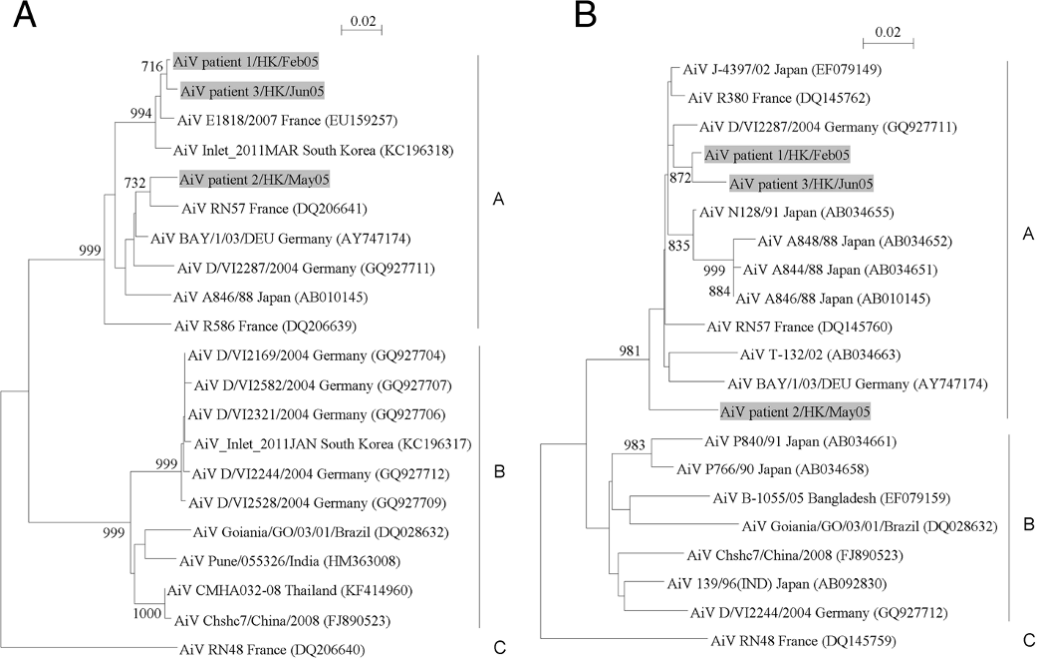
**Phylogenetic trees of AiV strains from 3 patients. (A)** Sequence for 295 nucleotide position in VP1 gene was included in the analysis. **(B)** Sequence for 342 nucleotide positions in partial 3CD region was included in the analysis. The trees are inferred by the neighbor-joining method with bootstrap value calculated from 1000 trees. The scale bar indicates the estimated number of substitutions of 50 nucleotides as indicated. The AiV strains detected in the present study are highlighted in grey.

For the genotyping of AiV, the partial VP1 capsid gene and 3CD region of all 3 AiV strains were amplified and sequenced. A phylogenetic analysis of nucleotide sequences of the partial VP1 gene of AiV showed that 2 AiV strains (AiV patient 1/HK/Feb05 and AiV patient 3/HK/Jun05) were closely related and formed a clade distinct from another strain AiV patient 2/HK/May05. Nevertheless, these 3 AiV strains clustered with genotype A strains identified in other countries (Figure 4A). This was consistent with the phylogenetic result using nucleotide sequences of the partial 3CD region of AiV (Figure 4B). These findings suggested that only one genotype, genotype A, of AiV was detected in Hong Kong in 2005. For the AiV-positive patient with multiple detections, the partial VP1 and 3CD sequences of AiV strains shed from the same patient were identical. For the genotyping of SalV, the partial VP1 gene and 3CD region of the SalV strain detected in this study could not be amplified, thus the genotype of this strain cannot be determined.

## Discussion

The present study represented the first to report the detection of HPeV, AiV and SalV in fecal samples from children with gastroenteritis in Hong Kong. HPeVs were distributed globally and found to be associated with acute gastroenteritis [23–28]. A number of studies have shown that the prevalence of HPeV infections in children with diarrhea ranged from 2% - 16.3% in various countries [19, 23, 24, 43], and it could be as high as 55% in China [28]. In the present study, the prevalence of HPeV infections was around 3%, which was similar to that in a study from Korea but lower than that reported in other studies [19, 23, 24, 28, 43]. HPeV infection was revealed to predominate in fall, which was in line with other studies in mainland China and Japan demonstrating that the highest detection rate of HPeV was noted during the autumn season [25, 44]. The reason for the high prevalence of HPeV during autumn is not known. Further studies are required to evaluate if temperature and relative humidity may be important determining factors. Of the 52 samples from 47 patients with HPeV infections, all showed positive in children who were ≤8 years old, with the majority (25/47, 53%) being younger than one year old. This indicated that HPeV infections mainly occurred in infants and younger children, which was consistent with the previous findings [23, 43]. In one case, a 1-year-old boy (patient 41) had a hospital-acquired HPeV-1 infection after an operation, suggesting that infection control measures may be required in controlling nosocomial transmission of HPeV. Another picornavirus, AiV, has been proposed to be a causative agent of gastroenteritis [21]. Several studies have demonstrated that the prevalence of AiV infections in pediatric patients with diarrhea ranged from 0.1% - 4.1% in America, Asia, Europe and Africa [31, 34, 37, 45–48]. The low prevalence of AiV infections (around 0.2%) in the present study was similar to that reported in a study from Canada [45]. Although the present study and most other studies showed low detection frequency of AiV in clinical specimens of patients with gastroenteritis, a high level of seroprevalence of AiV (>80%) in adults has been demonstrated [49], suggesting the widespread of human exposure to AiV during childhood. The high seroprevalence together with low detection rate of AiV in gastroenteritis indicated that AiV infections are usually asymptomatic or mild. A recently identified picornavirus, SalV, was found to be associated with gastroenteritis [40]. The prevalence of SalV infection (<0.1%) in this study was far lower than that reported in other studies [39, 41, 42]. In the only available study from China, SalV was detected in 9 (4.2%) of 216 diarrhea samples and 0 (0%) of 96 control samples [42]. The differences in the prevalence of HPeV, AiV and SalV infections among various countries were probably attributed to different sampling sources and detection methods (e.g. conventional RT-PCR, nested RT-PCR and real-time PCR), and variations in geographic and temporal conditions. While the present study aims to study the epidemiology of the novel picornaviruses instead of developing an improved diagnostic assay, it would be interesting to compare our detection method and other methods in future studies for their performance in diagnosis of HPeV, AiV and SalV infections.

The present data suggested that HPeV-1 was the predominant genotype among HPeVs, while genotype A was the predominant genotype among AiVs in Hong Kong. Phylogenetic analysis using the partial VP1 gene sequences of HPeV strains revealed that 23 (70%) of 33 typeable HPeV strains were closely related to HPeV-1 strains identified in other countries. In addition to HPeV-1, other HPeV genotypes, including types 3, 4, 5, 7 and 10, were detected in the present fecal samples. The low detection frequency of these 5 genotypes was also noted in previous studies showing that they were the rare HPeV genotypes identified in fecal samples from hospitalized patients [24, 26, 43]. To determine the genotype of AiV, the 3CD region of AiV was sequenced. Since capsid region is also commonly used for genotypic determination of other picornaviruses, we also sequenced the partial VP1 gene for phylogenetic analysis. The clustering pattern in the phylogenetic tree constructed using the partial VP1 gene sequences of AiV was in agreement with that using 3CD region (Figure 4). This demonstrated that genotype of AiV can be well determined by using either VP1 or 3CD sequences. The 3 AiV strains in this study clustered with other known genotype A strains, indicating that only AiV strains of genotype A were circulating in Hong Kong in 2005. However, we failed to determine the genotype of the SalV strain from patient 1/HK/Mar05, which may be due to low viral load in the samples and/or sequence mismatches between primers and viral gene sequences.

Co-detection of various diarrheal pathogens by PCR or culture previously was observed in fecal samples positive for HPeV in the present study. The frequency of co-detection in HPeV-positive fecal samples in this study (46.2%; 24/52) was in line with 2 previous studies from China showing the co-detection rate ranged from 52.3%-71.4% in fecal samples of children with acute gastroenteritis [44, 50], suggesting that co-detection is common for HPeV. Further studies are required to determine whether HPeV plays a causative role in these co-infections or act to exacerbate the disease caused by another pathogen. Besides, multiple virus detection was observed in 5 patients with HPeV infection in this study, in which persistent HPeV shedding for more than 2-week period was noted in fecal samples of patient 19 and patient 23. The prolonged shedding was probably due to the incomplete clearance of the virus by the immune system of the patients. Further investigation is warranted to examine the duration of HPeV shedding.

There are several limitations in this study. Firstly, the difference in epidemiological findings between the retrospective and prospective periods might be attributable to the different sample size (1440 and 268 respectively). Further study is warranted to collect more fecal samples for subsequent prospective analysis. Secondly, there is lack of data for the presence of other enteric viruses including enteric adenovirus, sapovirus, torovirus and astrovirus in the picornavirus-positive fecal samples, so the co-detection rate might be underestimated. Further investigation is required to determine if they are present in the samples. Thirdly, the inclusion of control groups in future may help examine the association between the picornaviruses (HPeV, AiV and SalV) and gastroenteritis.

## Conclusions

Emerging human picornaviruses including HPeV, AiV and SalV were detected in fecal samples of children with acute gastroenteritis in our locality. HPeV is the most prevailing virus with peak activity in fall in Hong Kong. The role of AiV and SalV in gastroenteritis remains uncertain because the number of AiV/SalV-positive samples was too small in the present study. Routine surveillance for these viruses in young children with gastroenteritis may better define their epidemiology and help prevent their transmission.

## Methods

### Patients and microbiological methods

A total of 1,708 fecal samples in this study were collected from hospitalized pediatric patients (age <18 years old) with gastroenteritis, which was defined as the development of acute diarrhea with 3 or more loose stools per day. All samples were obtained from three public hospitals in Hong Kong. A retrospective study during a 13-month period (November 2004 to August 2005 and August 2006 to October 2006) and a prospective study during a 12-month period (September 2012 to August 2013) were conducted. All fecal samples were tested for common bacterial diarrheal pathogens, rotavirus by antigen detection, norovirus by RT-PCR and HBoV by PCR [20]. The laboratory results of patients positive for HPeV, AiV and SalV were analyzed retrospectively. The study was approved by the Institutional Review Board of the University of Hong Kong/Hospital Authority Hong Kong West Cluster.

### RNA extraction

Viral RNA was extracted from fecal samples using EZ1 Virus Mini Kit v2.0 (QIAgen, Hilden, Germany). The RNA was eluted in 60 μl of AVE buffer and was used as the template for RT-PCR.

### RT-PCR for picornaviruses

RT was performed using random hexamers and the SuperScript III kit (Invitrogen, San Diego, CA, USA) as described previously [14–16]. PCR for HPeV, AiV and SalV was performed using two sets of primers designed by multiple alignments of 5′UTR nucleotide sequences of the corresponding picornaviruses (Table 2). Each PCR mixture (25 μl) contained cDNA, PCR buffer, 2 mM MgCl_2_, a 200 μM concentration of each deoxynucleoside triphosphate, and 1.0 U Taq polymerase (Boehringer, Mannheim, Germany). The mixtures were amplified by 50 cycles of 94°C for 1 min, 55°C for 1 min, and 72°C for 1 min, with a final extension at 72°C for 10 min. The amplified products were detected by agarose gel electrophoresis. Both strands of PCR products were sequenced twice with an ABI Prism 3730xl DNA Analyzer (Applied Biosystems, Foster City, CA, USA), using the PCR primers. The nucleotide sequences were compared to the corresponding sequences of other picornaviruses available in the GenBank.

**Table 2:** **Primers used in this study**

Virus	Forward primer sequence (5′-3′)	Reverse primer sequence (5′-3′)	PCR product size (Target)	Purpose
HPeV	CCYCTGGGSCCAAAAGSCA	GGTACCTYCWGGGCATCCTT	145 bp (5′UTR)	Screening of HPeV
AiV/SalV	CTGAGAMGRYGTTCCGCTGT	GACAATTAGCCCAGGSTCAGAT	215 bp (5′UTR)	Screening of AiV/SalV
HPeV	TCATGGGGTTCNCARATGGA	GATACCATAGTGYTTRTARAA	774 bp	Amplification of VP1 region
AiV	TCTTCTCCTTCTACCGCTTG	GAGGTGTAGGGGATGGAGAA	357 bp	Amplification of VP1 region
AiV	GCCAGTACAAGGACATGCGG	CGGTTGACGTTGACGCCAGG	381 bp	Amplification of 3CD region
SalV	CCCCRTCAACTTCCAGCAAA	ACACGAACGATRGAGGTGCT	482 bp	Amplification of VP1 region
SalV	GAGGGCACCGACCTGGATGC	TGGTTGATGAGAGAACCAAG	439 bp	Amplification of 3D region

### Phylogenetic analysis

To determine the genotype of HPeV, AiV and SalV detected in fecal samples, partial capsid and/or 3CD regions of these viruses were amplified and sequenced. Partial VP1 fragments of HPeV, AiV and SalV were amplified using three sets of primers designed by multiple alignments of VP1 nucleotide sequences of the corresponding picornaviruses (Table 2). Partial 3CD regions of AiV and SalV were amplified using two sets of primers designed by multiple alignment of 3C-3D nucleotide sequences of the corresponding picornaviruses (Table 2). Both strands of PCR products were sequenced twice with an ABI Prism 3730xl DNA Analyzer (Applied Biosystems, Foster City, CA, USA), using the PCR primers. The nucleotide sequences of the partial VP1 gene or 3CD region of the virus strains identified in the present study were compared to the corresponding sequences of other strains available in the GenBank. Phylogenetic tree construction was performed using neighbor-joining method with GrowTree using Kimura’s two-parameter correction, with bootstrap values calculated from 1000 trees (Genetics Computer Group, Inc.).

### Nucleotide sequence accession numbers

The partial VP1 nucleotide sequences of the HPeV and AiV strains and 3CD nucleotide sequences of the AiV strains have been lodged within the GenBank database under accession numbers KJ796868-KJ796906.

## Abbreviations

HPeV: Human parechovirus
AiV: Aichi virus
SalV: Salivirus
RT-PCR: Reverse transcription polymerase chain reaction
UTR: Untranslated region
HBoV: Human bocavirus
HK: Hong Kong.
